# FMDV Leader Protein Interacts with the NACHT and LRR Domains of NLRP3 to Promote IL-1β Production

**DOI:** 10.3390/v14010022

**Published:** 2021-12-23

**Authors:** Sk Mohiuddin Choudhury, Xusheng Ma, Yuanyuan Li, Xiaofeng Nian, Zhikuan Luo, Yonghua Ma, Zixiang Zhu, Fan Yang, Weijun Cao, Haixue Zheng

**Affiliations:** 1 State Key Laboratory of Veterinary Etiological Biology, National Foot and Mouth Disease Reference Laboratory, Key Laboratory of Animal Virology of Ministry of Agriculture, Lanzhou Veterinary Research Institute, Chinese Academy of Agricultural Sciences, Lanzhou 730046, China; mohiuddin.bau.vet786@gmail.com (S.M.C.); maxushengtt@163.com (X.M.); cuicuinian@163.com (X.N.); luozhikuan@yeah.net (Z.L.); zhuzixiang@caas.cn (Z.Z.); yangfan@caas.cn (F.Y.); caoweijun@caas.cn (W.C.); 2 Department of Animal Medicine, Gansu Agricultural University, Lanzhou 730070, China; lyy13321320240@163.com (Y.L.); mayh517@163.com (Y.M.)

**Keywords:** foot-and-mouth disease virus, inflammation, NLRP3, leader protein, NF-κB, ion channel

## Abstract

Foot-and-mouth disease virus (FMDV) infection causes inflammatory clinical symptoms, such as high fever and vesicular lesions, even death of animals. Interleukin-1β (IL-1β) is an inflammatory cytokine that plays an essential role in inflammatory responses against viral infection. The viruses have developed multiple strategies to induce the inflammatory responses, including regulation of IL-1β production. However, the molecular mechanism underlying the induction of IL-1β by FMDV remains not fully understood. Here, we found that FMDV robustly induced IL-1β production in macrophages and pigs. Infection of Casp-1 inhibitor-treated cells and NOD-, LRR- and pyrin domain-containing 3 (NLRP3)-knockdown cells indicated that NLRP3 is essential for FMDV-induced IL-1β secretion. More importantly, we found that FMDV L^pro^ associates with the NACHT and LRR domains of NLRP3 to promote NLRP3 inflammasome assembly and IL-1β secretion. Moreover, FMDV L^pro^ induces calcium influx and potassium efflux, which trigger NLRP3 activation. Our data revealed the mechanism underlying the activation of the NLRP3 inflammasome after FMDV L^pro^ expression, thus providing insights for the control of FMDV infection-induced inflammation.

## 1. Introduction

Foot-and-mouth disease (FMD) is a highly contagious acute and economically devastating viral disease of cloven-hoofed animals caused by the foot-and-mouth disease virus (FMDV). FMDV infection can damage host tissues and induce an inflammatory response, such as fever, blisters and ulcers in the mouth, hoof, nose, or breast, ulcers in the throat, trachea, bronchus and gastric mucosa, and hemorrhagic inflammation in the small intestine and large intestine mucosa [1,2]. The FMDV genome is a positive-stranded RNA and belongs to the genus Aphthovirus in the family Picornaviridae [3]. FMDV encodes four structural proteins (VP1, VP2, VP3, and VP4) and eight nonstructural proteins (L^pro^, 2A, 2B, 2C, 3A, 3B, 3C, and 3D) [4,5]. FMDV or FMDV proteins have been described to induce innate immune response. For example, FMDV infection causes inflammatory response in cloven-hoofed animals, such as pigs, cattle, and sheep [6]; cytokine mRNAs are significantly increased in FMDV-infected cattle [7]. FMDV VP3 protein interacts with TLR4 (Toll-like receptor 4, TLR4) to promote TLR4-mediated inflammatory response [6]. FMDV 2B protein also activates NLRP3 inflammasome to induce the IL-1β production [8]. Previous reports have demonstrated that proinflammatory cytokines are essential components of host innate immunity, which have a strong effect in virus defense [9,10].

The innate immune system is the first-line defense to viral invasions [11,12]. Pattern recognition receptors (PRRs), such as Toll-like receptors (TLRs) and NOD-like receptors (NLRs), recognize the virus during infection and trigger a protective immune response that eliminate the pathogens [13,14]. NLRs are stimulated by PAMPs (pathogen-associated molecular patterns) or DAMPs (damage-associated molecular patterns) and proinflammatory cytokines are induced to further antagonize viral infection. Increasing characteristic proinflammatory cytokine IL-1β levels is an important method of defending against invading viral infection. IL-1β is a well-studied proinflammatory cytokine in the IL-1 superfamily that mediates inflammatory response regulation during viral infection [15]. The NLRP3 inflammasome-mediated IL-1β production requires priming and activation steps to assemble. The priming signal is initiated by pattern recognition receptors (PRRs) and induces nucleus factor-κB (NF-κB) activation, which causes the transcription of the NLRP3 and IL-1β genes [16]. PAMP- or DAMP-induced PRR activation causes pro-IL-1β transcription and inactivates pro-IL-1β production [17]. The assembly of the complex of NLRP3 and ASC is the second signal of NLRP3 inflammasome activation. Upon host cell stimulation with DAMPs, NLRP3 and ASC oligomers form a multiple-protein complex as an initiator that recruits and facilitates pro-Casp-1 cleavage into the active Casp-1 enzyme. Eventually, the auto catalytic activation of Casp-1 leads to the splitting of pro-IL-1β into a bioactive form to trigger inflammation [17]. Three models of the stress-induced second signal of NLRP3 inflammasome activation are widely supported: (1) the ion channel model [18,19]; (2) the lysosomal rupture model [20]; and (3) the reactive oxygen species (ROS) model [21]. The ion channel model facilitates NIMA-related kinase 7 (Nek7)–NLRP3 binding and triggers the assembly of NLRP3 and apoptosis-associated speck-like protein with a CARD domain (ASC) [22,23]. Recently, studies have found evidence that NEK7 is involved in the NLRP3 inflammasome activation in several pathways, for instance, lysosomal destabilization, NF-κB signaling, ROS signaling, and potassium efflux [24]. Nek7 is an essential mediator of NLRP3 activation downstream of potassium efflux.

Different kinds of viruses can induce NLRP3 inflammasome-mediated inflammatory responses. For example, severe acute respiratory syndrome coronavirus-2 (SARS-CoV-2) infection triggers the NLRP3 inflammasome in COVID-19 patients [25]. The influenza virus activation of the NLRP3 inflammasome is dependent on lysosomal maturation and ROS [26]. Hepatitis C virus (HCV) RNA triggers IL-1β transcription through Toll-like receptor 7 (TLR7) signaling, and the HCV core protein activates the NLRP3 inflammasome to drive IL-1β secretion [27]. The enterovirus 71 (EV 71) 3D protein associates with NLRP3 to promote the assembly of the NLRP3 inflammasome multiple-protein complex [28]. Zika virus NS5 interacts with NLRP3 to facilitate NLRP3 inflammasome formation [29], and FMDV 2B activates NLRP3 inflammasome-mediated IL-1β production through elevation of intracellular ion [8].

Since FMDV has been demonstrated to induce the NLRP3 inflammasome-mediated inflammatory responses, the mechanisms of FMDV-induced NLRP3-mediated IL-1β production are still not completely understood. In the present study, we found a mechanism by which FMDV activates IL-1β production in pigs by promoting NLRP3 inflammasome assembly. The data showed that FMDV induced IL-1β production in vivo and in vitro. Importantly, we found that FMDV L^pro^ interacts with the NACHT and LRR domains of NLRP3 to promote NLRP3-ASC assembly and IL-1β production. Furthermore, L^pro^ induced calcium influx and potassium efflux to activate the NLRP3 inflammasome in the second step. Our study reveals that FMDV infection promotes IL-1β production by interacting with NLRP3 inflammasome via the ion channels and NF-κB mediated pathway, thus providing a theoretical basis for the control of FMDV-induced inflammation.

## 2. Materials and Methods

### 2.1. Animals, Clinical Specimens, and Blood Samples

Pigs (Landrace type, 3 months of old) were infected with FMDV (FMDV O/Mya/98, TCID_50_ = 1 × 10^7^) by intramuscular injection. After 5 days, serum and tissues (liver and kidney) of FMDV-infected pigs (Landrace type, n = 9) and healthy pigs (Landrace type, n = 9) were collected from the Animal Biosafety Level-3 (ABSL-3) lab of the Lanzhou Veterinary Research Institute (Lanzhou, Gansu, China) for cytokine detection. The FMDV was kept in the National Foot and Mouth Diseases Reference Laboratory, Lanzhou Veterinary Research Institute, Chinese Academy of Agricultural Sciences. FMDV replicated in BHK-21 cells for 7 passages. The animal study was directed according to the declaration of World Medical Assembly (WMA) and approved by the Lanzhou Veterinary Research Institute. Animal ethics were strictly followed during the animal study (NO. LVRIAEC-2021-012).

### 2.2. Cells and Cultures

Porcine kidney (PK-15) (ATCC#CCL-33) and human embryonic kidney 293 (HEK293) (ATCC #CRL-1573) cells were purchased from the American Type Culture Collection (USA). PK-15 cells were cultured in minimum essential medium (MEM, Gibco, Waltham, MA, USA) supplemented with 10% heat-inactivated FBS, penicillin (100 U/mL), and streptomycin sulfate (100 µg/mL). Human embryonic kidney 293 (HEK293) cells were cultured in Dulbecco’s modified Eagle’s medium (DMEM, Gibco, Waltham, MA, USA) supplemented with 10% heat-inactivated FBS, penicillin (100 U/mL), and streptomycin sulfate (100 µg/mL). Pig BMDMs (bone marrow derived macrophages) were collected from 90-day-old pigs and cultured in RPMI-1640 in the presence of granulocyte macrophage colony-stimulating factor with 10% heat-inactivated FBS for five days. The culture medium was replaced every other day. All cells were maintained at 37 °C with 5% CO_2_.

### 2.3. Viruses and Infection

FMDV O/Mya/98 was maintained by the National Foot and Mouth Diseases Reference Laboratory, Lanzhou Veterinary Research Institute, Chinese Academy of Agricultural Sciences. Virus propagation was conducted in Animal Biosafety-level 3 (ABSL-3) containment facility at the Animal, Lanzhou Veterinary Research Institute, Chinese Academy of Agricultural Sciences. On day 3 after passaging adherent BHK-21 cells, the medium was changed and the cells were infected with FMDV O/Mya/98 at a multiplicity of infection (MOI) of 1, 1.5, 2 or 2.5. Supernatants from infected cultures were harvested at 12, 16 and 24 h post-infection (hpi). FMDV titers were determined in the adherent BHK-21 cells via an endpoint titration using the Spearman–Kärber calculation and were presented as the tissue culture infective dose affecting 50% of the cultures (TCID_50_) per mL [30,31]. Each experiment was carried out in triplicate. Sendai virus (SeV) was maintained by our laboratory. For the FMDV infection confocal assays, PK-15 cells were infected with FMDV at a multiplicity of infection (MOI) of 2.5 at 37 °C for 12 h.

### 2.4. Reagents and Antibodies

Nigericin and LPS were purchased from InvivoGen Biotech Co., Ltd. (San Diego, CA, USA). Mouse anti-FLAG, mouse anti-myc, mouse anti-β-actin, mouse anti-HA, rabbit anti-IL-1β, rabbit anti-NLRP3, rabbit anti-Casp-1, and rabbit anti-ASC monoclonal antibodies were purchased from Cell Signaling Technology (Danvers, MA, USA). Horseradish peroxidase-conjugated anti-rabbit antibody or anti-mouse antibody were purchased from ZSGB-BIO, Lnc. The translation inhibitor cycloheximide (CHX) was purchased from APEXBIO (Houston, TX, USA). Lipofectamine 2000 was purchased from invitrogen (Waltham, MA, USA). 

### 2.5. Plasmids Construction

Myc-tagged NLRP3, ASC, Casp-1, and myc-tagged NLRP3 mutants (PYD, NACHT, and LRR) plasmids were generated by inserting porcine full-length NLRP3 (Genbank number: NM_001256770.2), ASC (Genbank number: XM_003124468.5), Casp-1 (Genbank number: NM_214162.1) and myc-tagged NLRP3 mutants (PYD, NACHT, and LRR) cDNA fragments into the pcDNA3.1 vector (Invitrogen, Carlsbad, CA, USA). Encoding FMDV viral structural and nonstructural genes were amplified from the FMDV genome and cloned into the Flag-CMV-7.1 vector (Sigma-Aldrich, St. Louis, MO, USA) to construct plasmids expressing Flag-tagged viral proteins by using standard molecular biology techniques. Primers used in this study for RT-PCR and the PCR primers of FMDV viral genes are shown in Table 1 and Table 2. NF-κB and pRL-TK reporter plasmids were kindly provided by Shu Hong Bing’s Lab, Wuhan University, China. All constructed plasmids were analyzed and verified by DNA sequencing.

### 2.6. Lentivirus Production and Infection

The target sequence of porcine NLRP3 shRNA was 5′-GGTGACCTCATATGACTAA-3′. Annealed short hairpin RNA (shRNA)-synthesized cDNA fragments corresponding to the cDNAs of swine NLRP3 genes were digested with BamHI and EcoRI and ligated into the pLVX vector (HANBIO, Shanghai, China), which was named lentivirus expressing NLRP3 shRNA (shNLRP3). A non-effective shRNA cassette in the pGFP-C-shLenti shRNA vector plasmid was used as the negative control and purchased from ORIGENE (Rockville, MD, USA). The lenti vector encoding shNLRP3 was transfected into HEK293 cells together with psPAX2 and pMD2.G with Lipofectamine 2000 (invitrogen, Waltham, MA, USA). Culture supernatants were harvested at 48 and 72 h, then filtered with a 0.45 μm filter, and centrifuged at 72,000× *g* for 120 min at 4 °C. PK-15 cells were infected with supernatants containing lentiviral particles. The shRNA knockdown efficiency was assessed by Western blot analysis. The IL-1β level and IL-1β relative mRNA levels in infected PK-15 cells were measured by ELISA (ELISA kit from RayBiotech, Inc., Catalog #: ELP-IL1b) and qRT-PCR (List of primers are given in Table 1).

### 2.7. Coimmunoprecipitation Assay (Co-IP)

PK-15 cells were cultured in 10 cm^2^ plates and transfected by the plasmids of L (5 μg) together with NLRP3 or NLRP3 mutants (5 μg) with Lipofectamine 2000 (invitrogen, Waltham, MA, USA). After 24 h, the cells were collected and lysed in 0.8 mL of lysis buffer (20 mM Tris-pH 7.5), 150 mM NaCl, 1% Triton X-100, 1 mM EDTA, 10 mg/mL aprotinin, 10 mg/mL leupeptin, and 1 mM PMSF). Lysates were incubated with 0.3 µL of indicated antibody or control IgG and 50 µL of G-Sepharose (GE Healthcare, Chicago, IL, USA) for 6–8 h. The Sepharose beads were washed three times with 1 mL of lysis buffer containing 500 mM NaCl. The samples were centrifuged, and replaced the wash buffer. Then the samples added 50 µL elution buffer. After boiling for 5 min, the precipitates were separated by SDS-PAGE.

### 2.8. Western Blot Analysis

The target proteins were resolved by SDS-PAGE and transferred to an Immobilon-P membrane (Millipore, Burlington, MA, USA) for Western blotting. The membrane was blocked in 5% skim milk for 1 h at room temperate and incubated with sufficient anti-myc primary (1:3000) at 4 °C overnight. The membrane was washed with TBST 5 times and incubated with horseradish peroxidase conjugated anti-mouse and anti-rabbit antibody assecondary antibodies (1:10,000) at room temperature for 1 h. Enhanced reacting chemiluminescence (Thermo, Waltham, MA, USA) was used to visualize antibody-antigen complexes. The results were analyzed by image lab software.

### 2.9. Confocal Microscopy

Nunc glass-bottom dishes were used to culture PK-15 cells. After 24 h of transfection with Lipofectamine 2000, the cells were fixed with 4% paraformaldehyde for 30 min. Subsequently, the cells were permeabilized with 0.1% Triton X-100 for 15 min. Then, the cells were incubated in 5% BSA for 15 min at 4 °C. The cells were incubated with primary antibody overnight and secondary antibody (Alexa Fluor 488- or 594-conjugated secondary antibody) for 1 h. The images were acquired with a laser-scanning confocal microscope (LSCM, Leica SP8, Solms, Germany).

### 2.10. RNA Extraction and RT-PCR

In a homogenizer, tissue samples were homogenized in trizol reagent (1 mL per 50–100 mg of tissue). Cells were lysed directly on the culture dish. A dose of 1 mL of the TRIzol reagent per 60 mm of culture plate surface area was used. After adding the reagent, the cell lysate was passed several times through a pipette to form a homogenous lysate. To ensure complete dissociation of nucleoprotein complexes, samples were allowed to stand for 5 min at room temperature. Then, 0.2 mL of chloroform was used per ml of TRIzol reagent. Samples were covered tightly, shaken vigorously for 15 s, and allowed to stand for 2–15 min at room temperature. The resulting mixture was centrifuged at 12,000× *g* for 15 min at 2–8 °C. Centrifugation separates the mixture into 3 phases: a red organic phase (containing protein), interphase (containing DNA), and a colorless upper aqueous phase (containing RNA). After that, RNA was isolated/separated using standard TRIzol reagent protocol.

cDNA was synthesized using Oligo dT Primer (50 μM), dNTP Mixture (10 mM each), template RNA, RNase Free dH2O. The mixture was incubated for 5 min at 6 °C, then cooled immediately on ice. After that, the reaction mixture was prepared in a total volume of 20 μL using template RNA Primer Mixture (from step 2)—10 μL, 5X PrimeScript Buffer—4 μL, RNase Inhibitor (40 U/μL)—0.5 μL (20 U), PrimeScript RTase (200 U/μL)—1.0 μL (200 U), and RNase Free dH2O was added to make the final volume 20 μL. It was mixed gently, and the reaction mixture was incubated at 42 °C for 60 min. Then the enzyme was inactivated by incubating at 95 °C for 5 min, and then the mixture was cooled on ice. The PCR mixture was prepared using TB Green Premix Ex Taq II (Tli RNaseH Plus, Cat# RR820A) (2X)—10 μL, PCR Forward Primer (10 μM)—0.8 μL, PCR Reverse Primer (10 μM)—0.8 μL, ROX Reference Dye (50X)—0.4 μL, template-2 μL, sterile purified water-6 μL to prepare the final volume 20 μL. A master mix was made with at least 10% more than the total volume needed for the total number of reactions to account for pipetting error. The PCR protocol is described below for BIO-RAD CFX96. Step 1—Initial Denaturation-95 °C for 30 s, Step 2—PCR-Goto: 40 cycles; 95 °C for 5 s, 60 °C for 30 s, Step 3—Melt curve. M-MLV reverse transcriptase (Promega, Madison, WI, USA) and random hexamer primers (Takara, Japan) were used to prepare cDNA. The generated cDNA was used as a template for FMDV RNA and cellular mRNA host expression. Real-time PCR (RT-PCR) was performed to measure the abundance of different mRNAs using Mx3005P qPCR (Agilent Technologies, Santa Clara, CA, USA) and SYBR Premix ExTaq reagents (Takara, Japan). The data were normalized to GAPDH expression. The 2^−ΔΔCt^ method was used to calculate the relative expression of mRNA. Primers used for qPCR/RT-PCR are mentioned in Table 1 and the PCR primers of FMDV viral genes are shown in Table 2.

### 2.11. Luciferase Reporter Assays

PK-15 cells (1 × 10^5^) were seeded in 48-well plates and Lipofectamine 2000 was used to transfect 100 ng of NF-κB reporter plasmid and 20 ng of pRL-TK into the cells. At 24 h, the cells were then mock-treated or infected with SeV/FMDV for 16 h. According to the manufacturer’s protocol, the luciferase activity was measured using the Dual-Luciferase Reporter Assay System (Promega, Madison, WI, USA). The data represent relative firefly luciferase activity normalized to the Renilla luciferase activity. Cell lysates were further used in Western blotting to evaluate protein expression. Three independent assays were conducted in the experiments.

### 2.12. ASC Oligomerization

The cell lysate supernatants were combined with SDS charging buffer to evaluate the Western blots using an ASC antibody. The pellets were washed three times with PBS, and after 30 min, they were connected with fresh DSS (2 mM, Sigma, St. Louis, USA). The related pellets were centrifuged and mixed for Western blot analysis using SDS loading buffers.

### 2.13. Enzyme-Linked Immunosorbent Assay (ELISA)

To detect the level of IL-1β in sera, organs, and cultured medium supernatants, ELISA kits were purchased from RayBiotech, Catalog #: ELP-IL1b (Peachtree Corners, GA, USA).

### 2.14. Mature IL-1β Measurement:

The supernatant (1 mL) of cultured cells was collected in cryogenic vials (Corning) and stored frozen at −80 °C for 24 h. A rotational vacuum concentrator (Christ-Alpha 1–2 LD plus) was used to lyophilize the samples, which were then dissolved in 100 μL phosphate-buffered saline (PBS) and mixed with sodium dodecyl sulfate (SDS) loading buffer for Western blot analyses using antibodies against mature IL-1β (Asp116 1:500; Cell Signaling).

### 2.15. Reagent Treatment

For the positive control sample of IL-1β production, the cells were first stimulated with LPS (60 ng/mL) for 8 h, then treated with Nigericin (2 µM) for 2 h, and then the supernatant or cells were collected for subsequent related experiments.

For the Caspase-1 inhibition experiment, the cells were first stimulated with LPS (60 ng/mL) for 8 h, then treated with Nigericin (2 µM) for 2 h, and then theVX-765 (10 µM) added into medium for 1 h, the supernatant or cells were collected for subsequent related experiments. For the translation inhibition experiment, the cells were stimulated with 2 μM Nigericin for 2 h primed with LPS for 8 h, then the cells treated with 100 μM CHX (10 µg/mL) for 1 h, the supernatant or cells were collected for subsequent related experiments.

### 2.16. Statistical Analysis

All tests were reproducible, and all experiments were performed independently at least three times. Sample variation was determined using Tukey’s post hoc test and analyzed by one-way ANOVA or *t*-test. Means are represented with histograms, with error bars representing the standard error of the mean (SEM), and *p* values < 0.05 were considered statistically significant.

## 3. Results

### 3.1. FMDV Infection Induces IL-1β Secretion in Pigs

**Figure 1:**
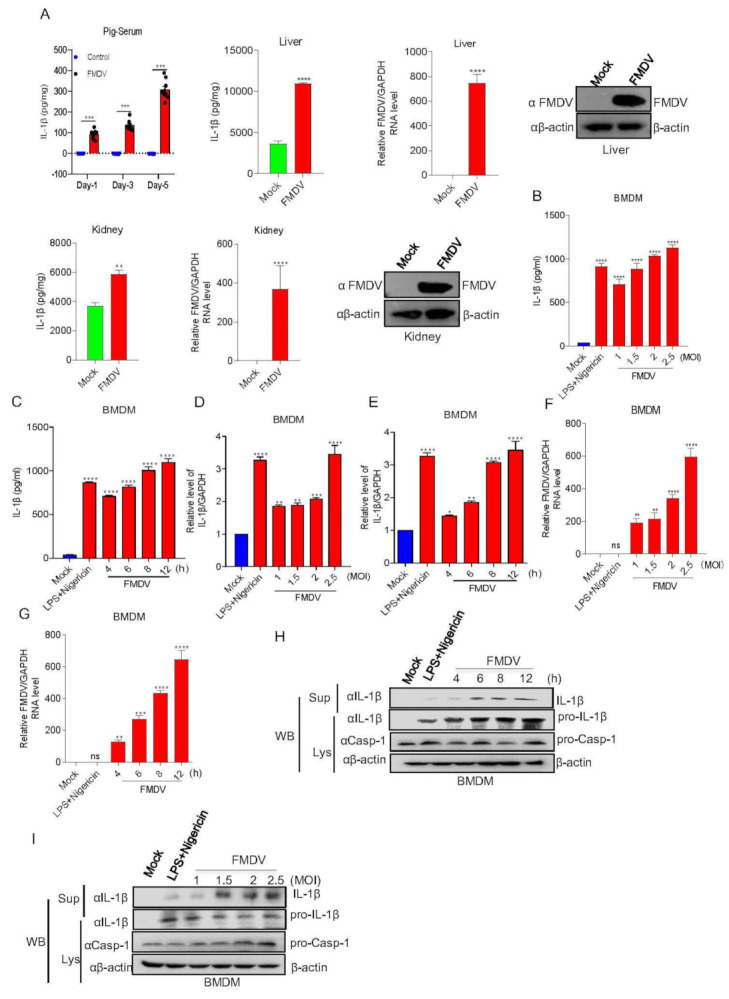
FMDV infection induces IL-1β secretion in pigs. (**A**) IL-1β levels of pig sera and organs with (n = 9) or without (n = 9) FMDV infection were detected by ELISA. Relative FMDV mRNA level and FMDV protein were detected by qPCR and Western blotting. Data shown are the mean ± SEM; ** *p* < 0.01, *** *p* < 0.001, **** *p* < 0.0001 (one-way ANOVA with Tukey’s post hoc test for Ai and *t*-test for Aii, Aiii). (**B**–**I**) BMDMs isolated from healthy pigs (3 months of age) were treated with 2 µM Nigericin for 2 h along with LPS for 8 h or infected with FMDV at MOIs of 1, 1.5, 2, or 2.5 for 12 h or MOIs of 2.5 for 4, 6, 8, or 12 h. The IL-1β levels in the medium were detected by ELISA (**B**,**C**). The IL-1β mRNA levels and FMDV mRNA levels at the indicated times and MOIs were determined by qPCR (**D**–**G**). IL-1β (17 kDa) expression in the supernatants or pro-IL-1β (31 kDa) and pro-casp-1 (45 kDa) expression in the lysates were detected by Western blotting (**H**,**I**). The data shown are the mean ± SEM; * *p* < 0.05, ** *p* < 0.01, *** *p* < 0.001, **** *p* < 0.0001 vs. mock treatment (one-way ANOVA with Tukey’s post hoc test for Ai, (**B**–**G**)). All experiments were repeated three times with similar results. Data are representative of three independent experiments.

In order to determine the effect of FMDV on IL-1β production in the natural host, IL-1β secretion and production after FMDV infection in pigs and pig BMDMs were investigated. As shown in Figure 1A, from day 1 to day 5, IL-1β secretion in the serum of FMDV-infected pigs gradually increased and was significantly higher than that in uninfected pigs. The effect of FMDV-induced IL-1β secretion on pig responses was investigated in the tissues. The results shown in Figure 1A indicate that FMDV induced IL-1β secretion in the liver and kidney. IL-1β could be secreted and expressed at high levels in macrophages. The effect of FMDV on IL-1β transcription and secretion in the macrophages of pig was detected. As shown in Figure 1B–G, IL-1β secretion and mRNA transcription with positive control (LPS + Nigericin) and FMDV infection increased at different hours post-infection (hpi) or multiplicities of infection (MOIs) compared with the findings under the control conditions. We also demonstrated that mature IL-1β protein expression after Nigericin along with LPS (LPS + Nigericin) treatment and FMDV infection increased in the supernatants and cells lysate (Lys) of pig BMDMs compared with the control conditions (Figure 1H,I). These data indicated that FMDV stimulation could induce IL-1β transcription and secretion in pigs.

### 3.2. FMDV Activates the NLRP3 Inflammasome to Induce IL-1β Secretion in Pig Cells

**Figure 2:**
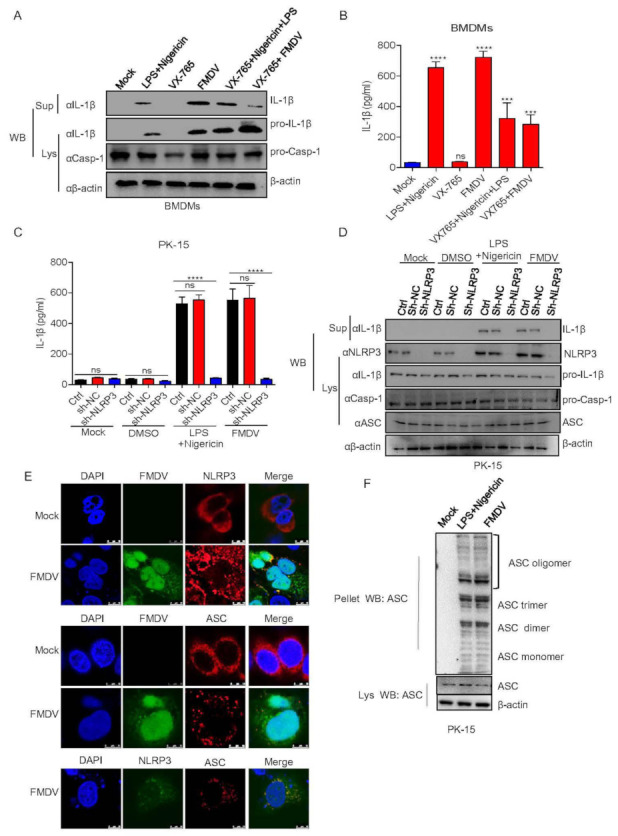
FMDV activates the NLRP3 inflammasome to induce IL-1β secretion in pig cells. (**A**,**B**) BMDMs were treated with 2 μM Nigericin (NLRP3 stimulator) for 2 h along with LPS for 8 h or VX-765 (casp-1 inhibitor) for 1 h or infected with FMDV at a MOI of 2.5 for 12 h or VX-765 together with Nigericin or VX-765 together with FMDV infection. IL-1β (17 kDa) expression in the supernatants or pro-IL-1β (31 kDa) and pro-casp-1 (45 kDa) expression in the lysates were detected by Western blotting (**A**). The IL-1β levels in the medium were determined by ELISA (**B**). (**C**,**D**) PK-15 cells stably expressing shRNAs targeting NLRP3 were generated and treated with DMSO or 2 µM Nigericin for 2 h and primed with LPS for 8 h or infected with FMDV at a MOI of 2.5 for 12 h. The IL-1β levels in the medium were determined by ELISA (**C**). IL-1β (17 kDa) expression in the supernatants or pro-IL-1β (31 kDa), ASC (22 kDa), NLRP3 (110 kDa) and pro-casp-1 (45 kDa) expression in the lysates were detected by Western blotting (**D**). (**E**) PK-15 cells were infected with FMDV at a MOI of 2.5 for 12 h. NLRP3, ASC and FMDV subcellular localization was assayed by confocal microscopy. The scale bar is 10 μm. (**F**) PK-15 cells were infected with FMDV at a MOI of 2.5 for 12 h or treated with 2 µM Nigericin for 2 h and primed with LPS for 8 h. ASC oligomerization with ASC primary antibody was detected by Western blotting. The data shown are the mean ± SEM; *** *p* < 0.001, **** *p* < 0.0001 vs. mock (**B**) or Ctrl (**C**) treatment (one-way ANOVA with Tukey’s post hoc test). All experiments were repeated three times with similar results. Data are representative of three independent experiments.

In order to determine whether FMDV induces IL-1β production associated with the NLRP3 inflammasome signaling pathway, first, maturation and secretion of IL-1β in BMDMs after the stimulation of FMDV and Casp-1 inhibitor were determined. As shown in Figure 2A,B, Nigericin along with LPS (positive control) and FMDV induced IL-1β secretion and protein expression while the Casp-1 inhibitor VX-765 inhibited this activation. The data suggested that FMDV induced IL-1β production associated with Casp-1 in pig cells. Furthermore, to address the effect of FMDV on NLRP3 inflammasome-mediated IL-1β production, we found that IL-1β secretion and protein maturation were impaired in PK-15 cells expressing shNLRP3 (short hairpin NLRP3, shNLRP3) after FMDV infection compared with control cells (Figure 2C,D). ASC speck formation is one of the direct indicators of NLRP3 inflammasome activation. As shown in Figure 2E, we determined that NLRP3 and ASC formed specks after FMDV infection but dispersed in uninfected cells, suggesting that FMDV induces inflammasome activity to facilitate NLPR3-ASC complex formation. ASC oligomer formation is another signal of inflammasome activation. Moreover, as detected in Figure 2F, FMDV and the positive control Nigericin along with LPS promoted ASC oligomerization in PK15 cells. All the above results suggested that FMDV induces IL-1β secretion and protein expression in pig cells through NLRP3 inflammasome activation.

### 3.3. FMDV Proteins Are Involved in NLRP3 Inflammasome Activation

**Figure 3:**
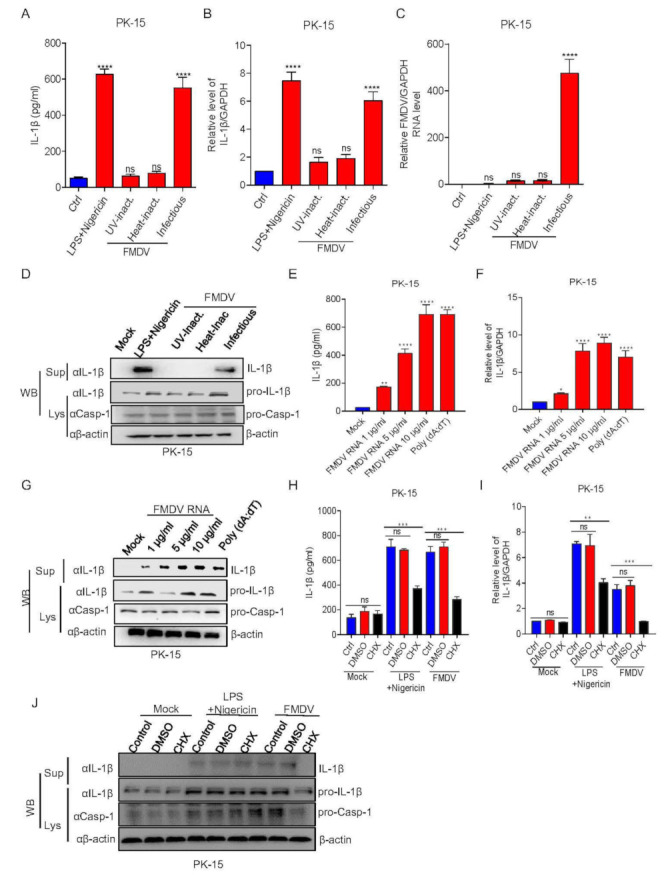
FMDV proteins are involved in NLRP3 inflammasome activation. (**A**–**D**) PK-15 cells were treated with 2 μM Nigericin for 2 h along with LPS for 8 h, inoculated with UV-inactivated FMDV or heat-inactivated FMDV, or infected with FMDV (MOI = 2.5) for 12 h. IL-1β levels in the medium were detected by ELISA (**A**) or IL-1β mRNA and FMDV mRNA levels were detected by qPCR (**B**,**C**). IL-1β (17 kDa) expression in the supernatants or pro-IL-1β (31 kDa) and pro-casp-1 (45 kDa) expression in the lysates were detected by Western blotting (**D**). (**E**–**G**) PK-15 cells were treated with Lipo, stimulated with Lipo plus poly (dA:dT), or treated with Lipo and transfected with 1, 5, or 10 µg/mL genomic RNA of FMDV for 24 h. IL-1β levels from the medium were detected by ELISA **(E)** and IL-1β mRNA levels were detected by qPCR (**F**). IL-1β (17 kDa) expression in the supernatants or pro-IL-1β (31 kDa) and pro-casp-1 (45 kDa) expression in the lysates were detected by Western blotting (**G**). (**H**–**J**) PK-15 cells were treated with 2 μM Nigericin for 2 h primed with LPS for 8 h, 100 μM CHX (translation inhibitor) for 1 h, or infected with FMDV (MOI = 2.5) for 12 h. IL-1β levels in the medium were detected by ELISA (**H**) and IL-1β mRNA levels were detected by qPCR (**I**). IL-1β (17 kDa) expression in the supernatants or pro-IL-1β (31 kDa) and pro-casp-1 (45 kDa) expression in the lysates were detected by Western blotting (**J**). The data shown are the mean ± SEM; * *p* < 0.05, ** *p* < 0.01, *** *p* < 0.001, **** *p* < 0.0001 (one-way ANOVA with Tukey’s post hoc test). All experiments were repeated three times with similar results. Data are representative of three independent experiments.

To investigate the step of FMDV infection that induces IL-1β production, PK-15 cells were stimulated with LPS + Nigericin, ultraviolet (UV)-inactivated FMDV, heat-inactivated FMDV, or infectious FMDV. We observed that LPS + Nigericin and FMDV infection induced IL-1β secretion and transcription but not UV-inactivated or heat-inactivated FMDV treatment (Figure 3A–C). Consistently, as shown in Figure 3D, the maturation protein of IL-1β was produced in the supernatants of PK-15 cells after FMDV infection, but not UV- or heat-inactivated FMDV treatment. These data suggested that FMDV infection and replication are integral for IL-1β production after FMDV is treated with UV or heat, which cause the virus to fail to replicate or infect. Additionally, to determine whether FMDV genomic RNA participates in IL-1β production, we detected IL-1β secretion after FMDV RNA transfection. As shown in Figure 3E, the FMDV RNA and inflammasome activator of poly(dA:dT) were involved in IL-1β secretion. Similarly, FMDV RNA and the positive control of poly(dA:dT) also induced pro-IL-1β transcription and IL-1β maturation protein expression (Figure 3F,G). A previous study showed that de novo translation does not affect NLRP3 inflammasome activation after ATP stimulation. On the contrary, EMCV induced IL-1β secretion was significantly inhibited after a protein synthesis inhibitor treatment of cycloheximide (CHX). The study suggested that EMCV viral proteins have an effect on NLRP3 inflammasome activation [32]. To determine whether FMDV viral proteins are involved in NLRP3 inflammasome activation, cells were treated with CHX or DMSO and then stimulated with LPS + Nigericin or FMDV. We observed that IL-1β production and transcription were induced after LPS + Nigericin and FMDV without CHX treatment, although CHX inhibited the production of IL-1β induced by LPS + Nigericin and FMDV (Figure 3H,I). Compared with the control, IL-1β cleavage protein also disappeared in the presence of CHX (Figure 3J). Taken together, these results showed that FMDV-induced IL-1β production required FMDV infection and replication. Meanwhile, FMDV viral proteins may involve in NLRP3 inflammasome-mediated IL-1β production.

### 3.4. FMDV Leader Protein Promotes NLRP3-Mediated IL-1β Production

**Figure 4:**
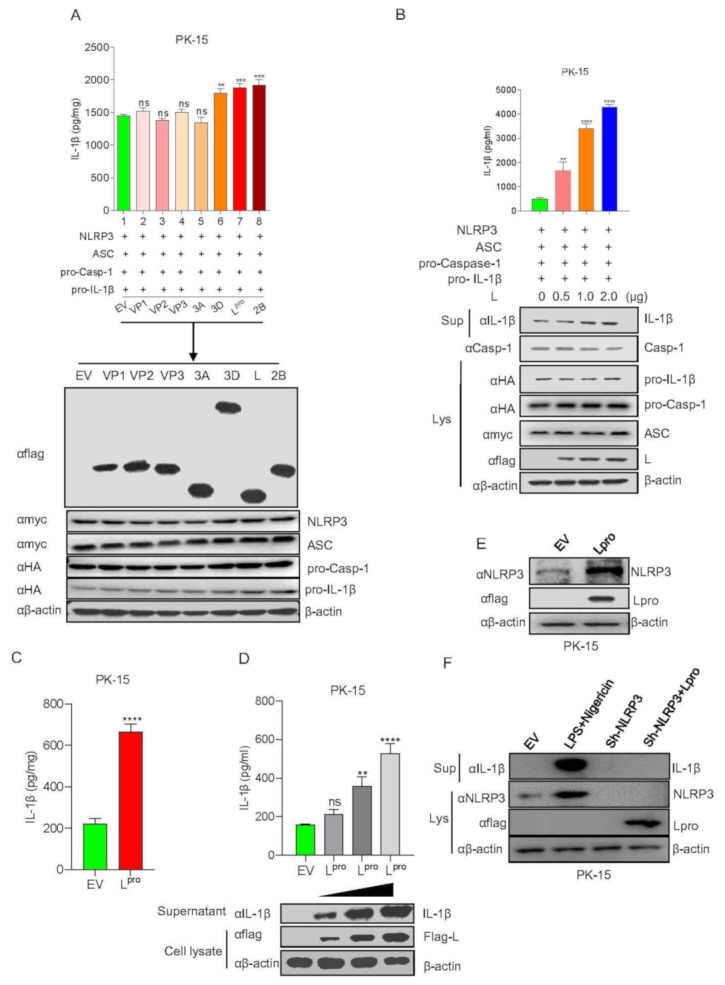
FMDV leader protein promotes NLRP3-mediated IL-1β production. (**A**) PK-15 cells were transfected with pcDNA 3.1 as an empty vector, Flag VP1, Flag VP2, Flag VP3, Flag 3A, Flag 3D, Flag L, Flag 2B co-transfected with Myc-NLRP3, Myc-ASC, HA-pro-caspase-1, HA-pro-IL-1β.IL-1β levels in the medium were detected by ELISA and plasmid expressions were detected by WB. (**B**) PK-15 cells were co-transfected with Myc-NLRP3, Myc-ASC, HA-pro-casp-1, and HA-pro-IL-1β and transfected with plasmids expressing Flag L of FMDV in a dose-dependent manner. IL-1β levels in the medium were detected by ELISA, and IL-1β (17 kDa) expression in the supernatants or mature Caspase-1 (20 kDa), pro-IL-1β (31 kDa), ASC (22 kDa), Flag L (24 kDa), and pro-casp-1 (45 kDa) expression in the lysates were detected by Western blotting. (**C**) PK-15 cells were transfected with empty vector pcDNA 3.1 and Flag-L of FMDV. IL-1β levels in the medium were detected by ELISA. (**D**) PK-15 cells were transfected with empty vector pcDNA 3.1 and plasmid-expressing Flag L of FMDV in a dose-dependent manner (1, 2, 4 µg). IL-1β levels in the medium were detected by ELISA; IL-1β (17 kDa) from supernatant and Flag L (24 kDa) from cell lysates were detected by WB. (**E**) PK-15 cells were transfected with empty vector pcDNA 3.1 and Flag-L. Cell lysates were subjected to SDS-PAGE and detected by Western blotting. (**F**) PK-15 cells were transfected with empty vector pcDNA 3.1, sh-NLRP3, Flag-L along with sh-NLRP3 or treated with LPS along with Nigericin. IL-1β (17 kDa) in the supernatants or, Flag L (24 kDa), NLRP3 (110 kDa) expression in the lysates were detected by Western blotting. The data shown are the mean ± SEM; ** *p* < 0.01, *** *p* < 0.001, **** *p* < 0.0001; ns, no significance (one-way ANOVA with Tukey’s post hoc test for (**A**–**C**), (**E**) or *t*-test for (**D**)). All experiments were repeated three times with similar results. Data are representative of three independent experiments.

A previous study showed that the expression of all NLRP3 inflammasome components of NLRP3, ASC, and pro-Casp-1 together significantly activate IL-1β secretion. To investigate the effect of FMDV protein on NLRP3-mediated IL-1β activation, each of the viral proteins was transfected into the NLRP3 inflammasome activation PK-15 cell system, which was established using NLRP3, ASC, pro-Casp-1, and pro-IL-1β co-expression. The results showed that the L^pro^, 2B, and 3D proteins increased IL-1β secretion (Figure 4A). Furthermore, we determined whether L^pro^ affected IL-1β secretion in a dose-dependent manner. L^pro^ was transfected into IL-1β production system cells at the indicated dose, and the results shown in Figure 4B indicated that L^pro^ promoted IL-1β secretion and IL-1β protein maturation in a dose-dependent manner. To confirm the effect of L^pro^ on IL-1β production in resting cells, we transfected L^pro^ alone without the NLRP3 inflammasome activation cell system and found that L^pro^ also induced IL-1β production (Figure 4C). Moreover, we determined whether L^pro^ increased IL-1β secretion in a dose-dependent manner in rest cells. In supernatant and cell lysate, the results indicate that L^pro^ promoted IL-1β secretion and IL-1β protein expression in a dose-dependent manner without the NLRP3 inflammasome activation cell system (Figure 4D). We also detected the effect of L on NLRP3 expression in PK-15 cells. As shown in Figure 4E, L^pro^ increased NLRP3 protein expression. Moreover, in order to determine whether L^pro^ affects NLRP3 inflammasome to increase IL-1βproduction, NLRP3 shRNA was transfected into PK15 cells, we found that L^pro^-triggered IL-1β maturation was impaired after NLRP3 knockdown (Figure 4F). Taken together, these results above suggested that L^pro^ activated NLRP3 inflammasome-mediated IL-1β production.

### 3.5. FMDV L^pro^ Interacts with the NACHT and LRR Domains of NLRP3 to Promote NLRP3 Inflammasome Assembly

**Figure 5:**
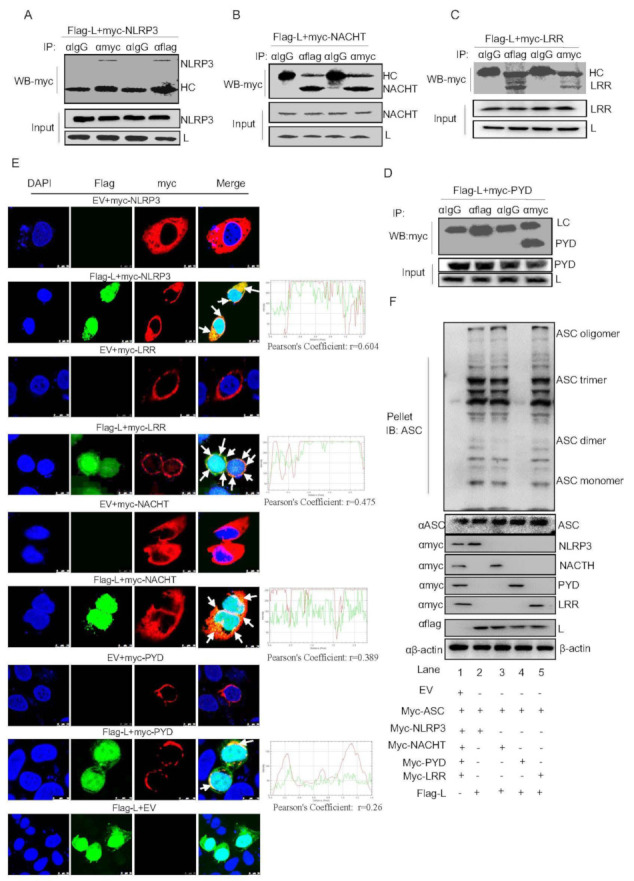
FMDV L^pro^ interacts with the NACHT and LRR domains of NLRP3 to promote NLRP3 inflammasome assembly. (**A**–**D**) PK-15 cells were transfected with the plasmids of Flag-L along with the Myc-NLRP3 (**A**), Myc-NACHT (**B**), Myc-LRR (**C**), or Myc-PYD (**D**). Cell lysates were subjected to IP using IgG, anti-Myc, or anti-Flag primary antibody to enrich Flag-Lpro, myc-NLRP3, Myc-NACHT, Myc-LRR or Myc-PYD proteins, respectively, and then detected the samples with anti-myc antibody. The whole cell lysate samples as input were subjected to Western blotting to detect plasmid expression. (**E**) PK-15 cells were transfected with EV+ Myc-NLRP3, Flag-L + Myc-NLRP3, EV+ Myc-LRR, Flag-L+ Myc-LRR, EV+ Myc-NACHT, Flag-L + Myc-NACHT, EV+ Myc-PYD, Flag-L + Myc-PYD, and EV+ Flag-L. Subcellular localization was observed by confocal microscopy. The white arrows indicate the co-localized area. The Pearson’s correlation coefficient was analyzed using the Image-J (Java 1.8.0_172) software. The scale bar is 10 μm. (**F**) PK-15 cells were co-expressed with empty vector pcDNA 3.1, myc-ASC, myc-NLRP3, Myc-NACHT, Myc-PYD, Myc-LRR or Flag L. Cell lysates and pellets were subjected to ASC oligomerization detection. All experiments were repeated three times with similar results. Data are representative of three independent experiments.

Next, we explored the mechanism by which L^pro^ facilitates NLRP3-mediated IL-1β production. We used the anti-IgG, anti-Flag, or anti-myc as the primary antibody to enrich Flag-L^pro^ or myc-NLRP3 protein, then used anti-myc for IB to detect whether L^pro^ interacts with NLRP3. The results showed that L^pro^ interacts with NLRP3 (Figure 5A). These results suggested that L^pro^ may combine with NLRP3 to affect IL-1β production. NLRP3 is composed of the PYRIN, NACHT, and LRR domains. Using co-IP, we demonstrated that L^pro^ interacts with the NACHT and LRR domains of NLRP3 but not the PYRIN domain (Figure 5B–D). Since L^pro^ targets NLRP3, the cellular localization of L^pro^ and NLRP3 was investigated via confocal microscopy and Pearson’s correlation coefficient analysis. We found that L^pro^ was mainly distributed in the cytoplasm and nucleus after expression (Figure 5E). Simultaneously, as shown in Figure 5E, L^pro^ colocalized with NLRP3 to promote speck formation. L^pro^ co-transfected with the NLRP3 domains of NACHT, PYD, or LRR, we found that most L^pro^ colocalized with NLRP3, NACHT, PYD, and LRR in the cytoplasm. To address the effect of L^pro^ on ASC oligomer formation, we observed that L^pro^ enhanced ASC oligomerization (Figure 5F). Moreover, the NACHT and LRR domain, but not the PYD domain, were involved in ASC oligomerization which facilitated by L^pro^. Taken together, these results showed that FMDV L^pro^ interacted with the NACHT and LRR domains of NLRP3 to promote the assembly of the NLRP3 inflammasome complex.

### 3.6. L^pro^ Activated the NLRP3 Inflammasome through an Ion Channel

**Figure 6:**
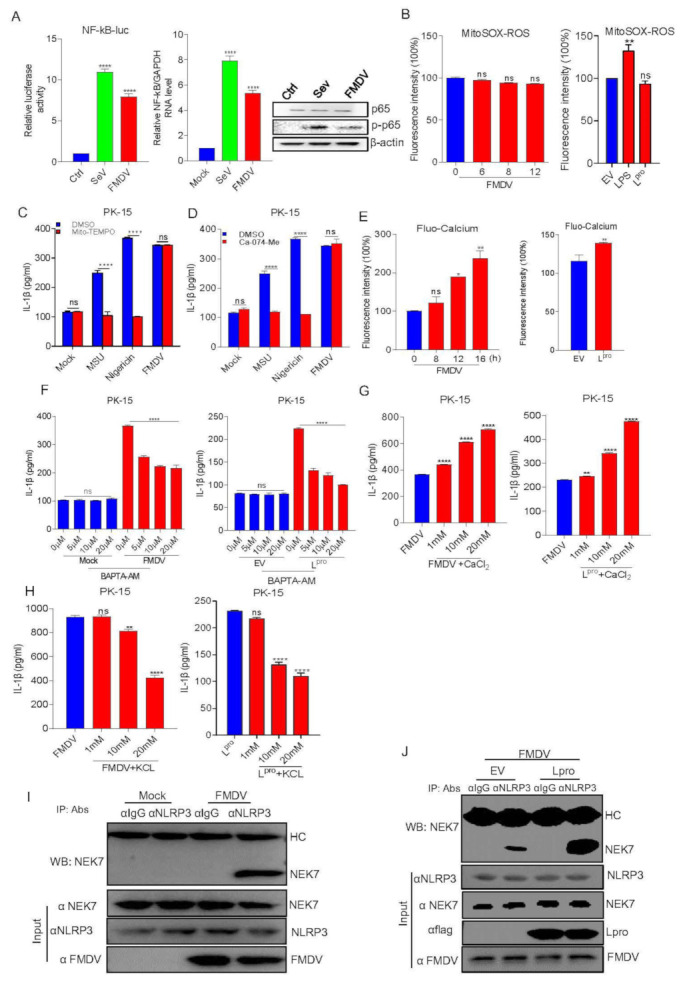
L^pro^ activated the NLRP3 inflammasome through an ion channel. (**A**) FMDV can induce NF-κB activation. PK-15 cells were transfected with the NF-κB luciferase reporter plasmid. After 24 h, cells were infected with SeV or FMDV, and controls were kept uninfected. The cell lysates were subjected to dual-luciferase assay, qPCR, and Western blot detection. (**B**) PK-15 cells were infected with FMDV or transfected with L or treated with LPS for 8 h as a positive control and also were treated with Mito-SOX for 10 min at 37 °C. The cell lysates were collected at the indicated time points, and the fluorescence intensity was analyzed by flow cytometry. Forty thousand cells were counted. (**C**,**D**) PK-15 cells were treated or untreated with MSU, Nigericin, and FMDV in the presence of DMSO, Mito-TEMPO, or Ca-074-Me. IL-β levels were determined by ELISA. (**E**) PK-15 cells were infected with FMDV or transfected with L and treated with Fluo-3 AM for 1 h at 37 °C. The cell lysates were collected at the indicated time points, and the fluorescence intensity was analyzed by flow cytometry. Forty thousand cells were counted. (**F**–**H**) PK-15 cells infected with FMDV or transfected with 3D were treated with BAPTA-AM, KCl, or CaCl_2_ at the indicated doses. IL-β levels were determined by ELISA. (**I**) PK-15 cells were infected with FMDV. Cell lysates were subjected to IP using IgG, anti-NLRP3, and anti-NEK7 primary antibody and detected with Western blotting using the NEK7 antibody. The whole cell lysate samples as input were subjected to Western blotting to detect the protein expression. (**J**) PK-15 cells were transfected with empty vector pcDNA 3.1 or L^pro^, then infected with FMDV. Cell lysates were subjected to IP using IgG, anti-NLRP3, and anti-NEK7 primary antibody and detected with Western blotting using the NEK7 antibody. The whole cell lysate samples as input were subjected to Western blotting to detect the protein expression. Data are presented as the mean ± SEM of triplicate measurements in three independent experiments. ns: not significant, * *p* < 0.05, ** *p* < 0.01, **** *p* < 0.0001, mock treatment or FMDV (one-way ANOVA with Tukey’s post hoc test). The results were analyzed by flow cytometric analysis with FlowJo software V10.

Since NF-κB activation is the initial signal for NLRP3 inflammasome activation, we then assessed the FMDV effect on NF-κB activation. As shown in Figure 6A, a luciferase reporter gene assay showed that FMDV infection significantly increased NF-κB promoter activation compared with the control. Consistently, NF-κB mRNA levels and p65 phosphorylation were also increased after FMDV infection. Together with Figure 1D,E, the results demonstrated that FMDV infection induced NF-κB activation and pro-IL-1β transcription.

NLRP3 inflammasome activation depends on the second signal. Thus, we tested the effect of FMDV and L^pro^ on the second signal of NLRP3 activation. Using fluorescence intensity detection, we observed that FMDV infection or L^pro^ expression did not affect ROS induction during Mito-SOX (Mitochondrial Superoxide Indicator, 5 μM MitoSOX for 10 min at 37 °C) treatment compared to that of the control (Figure 6B). Mito-TEMPO is an inhibitor of NLRP3 that inhibits ROS production, and monosodium urate (MSU) is an activator of the NLRP3 inflammasome. We observed that FMDV-infected cells had no effect on IL-1β production after Mito-TEMPO (500 μM) treatment while LPS + Nigericin- or MSU (2.5 mM)-induced IL-1β secretion was significantly inhibited after Mito-TEMPO treatment (Figure 6C). These data showed that FMDV activated NLRP3 independent of the ROS model. Cathepsin B protease from lysosomes is an indicator of NLRP3 inflammation. As shown in Figure 6D, FMDV and L^pro^ had no effect on FMDV-induced IL-1β production during treatment with Ca-074-Me (a specific cathepsin B inhibitor, 10 μM). This result suggested that FMDV infection-induced IL-1β secretion was also independent of the lysosome model. The ion channel model always focuses on cytosolic K^+^ efflux and Ca^2+^ influx, which activate NLRP3 inflammation. As shown in Figure 6E, the cells were stained with the calcium-dependent fluorescent dye Fluo-3 AM after FMDV infection or L^pro^ expression to test the intracellular Ca^2+^ levels. FMDV and L^pro^ stimulation significantly increased the intracellular Ca^2+^ levels. Consistently, FMDV infection- or L^pro^ expression-induced IL-1β secretion was significantly decreased in a dose-dependent manner after the cells were treated with the Ca^2+^ chelator BAPTA-AM (Figure 6F). We also found that IL-1β secretion was increased in FMDV-infected or L^pro^-expressing cells treated with CaCl_2_ at gradient concentrations (Figure 6G), thus further confirming the accuracy of the effect of FMDV infection or L^pro^ expression on ion channel-induced IL-1β production. Similarly, as shown in Figure 6H, FMDV-infection- or L^pro^-induced IL-1β secretion was inhibited after treatment with gradient concentrations of KCl. All the above results suggested that FMDV infection or L^pro^-induced IL-1β secretion was dependent on the ion channel model.

Nek7 is an essential mediator of NLRP3 activation downstream of potassium efflux. The results above proved that FMDV and L^pro^ induce ion channel-mediated NLRP3 activation, so we wondered whether FMDV and L^pro^ affect the regulation of NLRP3 activation by NEK7. PK-15 cells were transfected with L^pro^ or infected with FMDV. Then we assessed the effect of FMDV and L^pro^ on NLRP3–NEK7 complex formation, we found that FMDV induced NLRP3 and NEK7 interaction (Figure 6I). Meanwhile, FMDV infected with PK-15 cells after EV or L^pro^ transfection, we observed that L^pro^ facilitated NLRP3 and NEK7 complex formation compared with control (Figure 6J). Taken together, these results suggested that FMDV or L^pro^ activate NLRP3 inflammasome by promoting the formation of the complex of NEK-NLRP3.

## 4. Discussion

The innate immune system is important for the host to clear the invading viruses [33]. The inflammasome assembly is essential for the host innate immune response against pathogen infection. The NLRP3 inflammasome is one of the well-characterized inflammasomes. It activates the maturation of IL-1β. Two separate signals were required for NLRP3 inflammasome-mediated IL-1β production: the primary signal is relayed to recognize PAMPs or DAMPs by its PRRs, resulting in the induction of the pro-IL-1β transcription, while the second signal is involved in the activation of different inflammasomes, leading to the cleavage of pro-IL-1β into mature IL-1β. In this study, we proved that FMDV RNA induced IL-1β production. Since the genome of FMDV is a single-stranded positive-strand RNA, we suspect that FMDV RNA may have two effects when it is transfected into cells. The first is FMDV RNA induces NF-κB activation and then IL-1β transcription. Similar to HCV, only the 3′UTR of the RNA genome contained the crucial motif for IL-1β induction [34], second, we speculated that the FMDV RNA genome may also have the specific sequence or critical structure for FMDV RNA-induced IL-1β secretion. Most likely, the FMDV RNA genome activates NF-κB, but the mechanism needs to be further revealed. NLRP3 inflammasome activation is the secondary signal for IL-1β maturation [17]. Our research also showed that FMDV 2B, 3D and L^pro^ promoted NLRP3 inflammasome activation, which is inconsistent with the findings from a previous study by Xiaoying Zhi et al. [8], who showed that only the 2B protein of FMDV activated the NLRP3 inflammasome. We speculated that the discrepancy between the two findings is due to the different cells employed in the two studies, with the study by Xiaoying Zhi et al. using cell lines derived from mice and our study using natural host cell lines. Harry D. Dawson and D. Allen et al. performed in-depth comparisons of the conservation of homology and structural motifs of the orthologs of pigs, mice and humans and found that the overall similarity of proteins between humans and pigs was significantly higher than that between humans and mice [35]. For instance, AIM2-like receptors (ALRs) contain 13 family members in mice while IFI16 and myeloid cell nuclear differentiation antigen (MNDA) only have two members in pigs. Meanwhile, other studies found that RAW264.7 macrophages failed to process and release mature IL-1β to all caspase-1-associated secondary stimuli because they do not express ASCs [22,36]. Therefore, we hypothesized that the inflammatory response may differ between mouse cells and pig cells.

Furthermore, we tried to uncover the mechanism of NLRP3 inflammasome activation induced by L^pro^, we found that L^pro^ interacted with NLRP3 to promote NLRP3 inflammasome activation. Although L^pro^ was associated with NLRP3, we also did not find decreased protein expression or cleaved fragments of pro-caspase-1, suggesting that the effect of L^pro^ on the NLRP3 inflammasome activation is not dependent on the L^pro^ papain-like protease activity. Thus, L^pro^-induced NLRP3 inflammasome activation is a novel function. NLRP3 contains NACHT, LRR, and PYD domains. In the study, we demonstrated that L^pro^ interacted with NACHT and LRR domains, not PYD. Consistently, L^pro^ also promoted ASC oligomerization with NACHT and LRR domain, but not PYD. A study suggested that LRR domain is unnecessary for mouse NLRP3 inflammasome activation [37]. However, most of viruses use the viral proteins to interact with the LRR domain to activate/promote NLRP3 inflammasome, such as EV71 3D interacted with LRR domain of NLRP3 [28]; Zika virus NS5 protein binds NLRP3 by interacting with NACHT and LRR domains [29]; and PB1-F2 of Influenza A viruses binds to the PYD and LRR domain of NLRP3 [38], so we speculated that LRR domain may act as the NLRP3 receptor domain for ligands after viral infection. Without stimulator, NLRP3 protein is thought to be auto-repressed through interaction between the domains of NACHT and LRRs. L^pro^ interacted with both NACHT with the LRRs, it may destroy the resting state of NLRP3 and promote the activation of NLRP3 inflammasome.

Confocal microscopy revealed the distribution of L^pro^ in both the cytoplasm and nucleus, whereas it showed that NLRP3 is localized in the cytosol. Consistent with the detected interaction, L^pro^ colocalized with the NACHT and LRR domains of NLRP3. NLRP3 inflammasome activation requires ASC oligomerization formation, and our data showed that FMDV and L^pro^ increased ASC oligomerization, which also suggested that L^pro^ promoted NLRP3 inflammasome activation.

The second step of NLRP3 activation is supported by three models, including (1) the lysosomal rupture model, which is caused by lysosomal damage and cathepsin B release, which lead to NLRP3 activation; (2) the ROS model, which promotes K^+^ circulation to induce NLRP3 inflammasome activation; and (3) the ion channel model, which invigorates the efflux of K+ or influx Ca^2+^ in cells and ultimately activates the NLRP3 inflammasome [39]. A previous study showed that FMDV and its 2B protein induced a high intracellular concentration of Ca^2+^. Meanwhile, both FMDV and the FMDV 2B protein promoted K^+^ efflux [8]. Consistently, we also demonstrated that FMDV and FMDV L^pro^ can induce K^+^ efflux and Ca^2+^ influx to trigger NLRP3 activation. In this study, the mechanism of L^pro^-induced ion channel-mediated NLRP3 inflammasome activation is still not clear. We speculated that FMDV L^pro^ and NLRP3 bind to and may potentially further affect the ion channel-mediated NLRP3 inflammasome activation.

**Figure 7:**
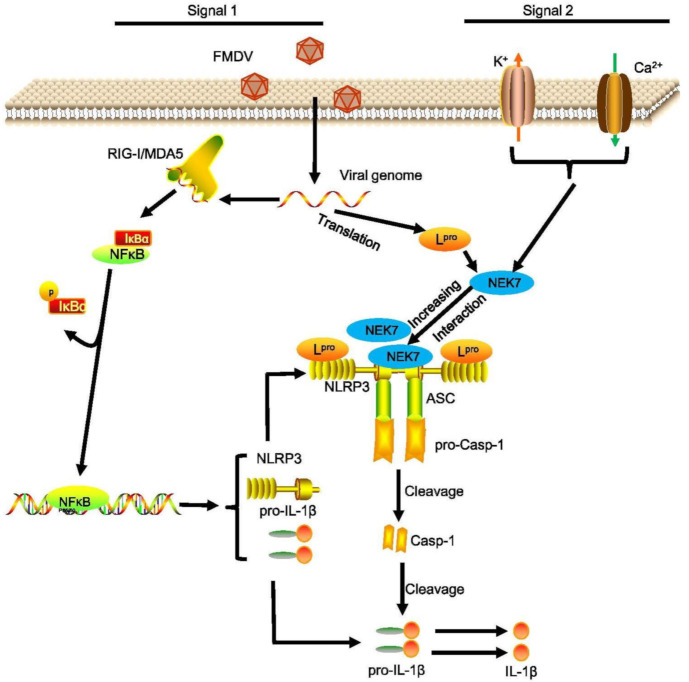
Proposed model for the L^pro^ mediated mechanism by which FMDV induces NLRP3-mediated inflammatory responses. First, upon FMDV infection, the FMDV RNA genome is recognized by MDA-5/RIG-I-like receptors and NF-κB-induced proinflammatory cytokine production. The L protein is translated by the FMDV genome during FMDV infection which induces NF-κB activation to increase pro-IL-1β transcription. Second, L^pro^ interacts with the NATCH and LRR domains of NLRP3 to facilitate the assembly of the L-NLRP3-ASC complex. Moreover, L^pro^ induces cytosolic K+ efflux and Ca^2+^ influx to promote NEK7–NLRP3 complex formation. Taken together, L^pro^ leads to the activation of NLRP3, ultimately causing IL-1β production and secretion. Then, IL-1β is involved in the initiation of proinflammatory signal transduction during FMDV infection.

Until now, the exact mechanism for NLRP3 inflammasome activation remains unclear. NLRP3 inflammasome induced by potassium (K^+^) efflux [40], increased intracellular calcium ions, decreased cellular cyclic AMP (cAMP) [41], lysosomal destabilization as well as reactive oxygen species (ROS) production [42,43]. Recently, the ion channel model facilitated a protein kinase NEK7 to act as a regulator binding with NLRP3 to trigger the assembly of NLRP3 and ASC [44]. Our data showed that FMDV induced the NLRP3 and NEK7 interaction. This suggested that the FMDV-induced NLRP3 inflammasome activation through the ion channel may be regulated by the NEK7 protein. Moreover, we found that L^pro^ facilitated NLRP3–NEK7 complex formation after FMDV infection. A previous study proposed that NLRP3 activation requires at least two steps: 1. NLRP3 binds with NEK7; 2. The conversion of NACHT needs to change from a resting status to an active conformation [45]. From our data, we estimated that L^pro^ may destroy the resting status of NLRP3 and promote FMDV-induced NEK7–NLRP3 interaction. The exact mechanisms need to be further studied. In conclusion, FMDV induced the production of IL-1β in natural hosts and cells. FMDV RNA, FMDV replication, and FMDV translation are involved in IL-1β secretion. We proposed a model for the L^pro^ mechanism by which FMDV induces NLRP3-mediated inflammatory responses. First, upon FMDV infection, the FMDV RNA genome is recognized by MDA-5/RIG-I-like receptors and NF-κB-induced pro-IL-1β of proinflammatory cytokine production. Second, the L protein during FMDV infection interacts with the NATCH and LRR domains of NLRP3 to facilitate the assembly of the NLRP3-ASC complex. Moreover, L^pro^ induces the ion channel to promote NEK7–NLRP3 interaction after FMDV infection. Taken together, L^pro^ leads to the activation of NLRP3, ultimately causing IL-1β production and secretion. Then, IL-1β is involved in the initiation of proinflammatory signal transduction during FMDV infection (Figure 7).

## Figures and Tables

**Table 1:** Primers used in this study for the RT-PCR.

Genes	Sense Primers (5′-3′)	Anti-Sense Primers (5′-3′)
hGAPDH-qRT	GAGTCAACGGATTTGGTCGT	GACAAGCTTCCCGTTCTCAG
P-GAPDH-qRT	ACATGGCCTCCAAGGAGTAAGA	GATCGAGTTGGGGCTGTGACT
mGAPDH-qRT	CCATGTTCGTCATGGGTGTGAACCA	GCCAGTAGAGGCAGGGATGATGTTC
h-p65-qRT	TGAACCGAAACTCTGGCAGCTG	CATCAGCTTGCGAAAAGGAGCC
h-IL-1β-qRT	GCAAGGGCTTCAGGCAGGCCGCG	GGTCATTCTCCTGGAAGGTCTGTGGGC
FMDV-qRT	ACTGGGTTTTACAAACCTGTGA	GCGAGTCCTGCCACGGA
m-IL-1β-qRT	GCACTACAGGCTCCGAGATGAAC	TTGTCGTTGCTTGGTTCTCCTTGT
P-caspase 1-qRT	GAAATACTCCGAAGAAGTCCCAGA	GACCCCTTGCTTCTCACCAC
P-PYCARD-qRT	TCAAGGGTCACAGACGTGGA	TTTGGTGGGGTTGGTGTG
h-NLRP3-qRT	AAGGGCCATGGACTATTTCC	GACTCCACCCGATGACAGTT
h-ASC-qRT	AACCCAAGCAAGATGCGGAAG	5′-TTAGGGCCTGGAGGAGCAAG-3′
h-Casp-1-qRT	TCCAATAATGCAAGTCAAGCC	GCTGTACCCCAGATTTTGTAGCA
P-IL-1β-qRT	GACGGGCTTTTGTTCTGCTT	GGACATGGAGAAGCGATTTGT
P-p65-qRT	GGAACACGATGGCCACTTG	AAGAGGACATCGAGGTGTATTTCAC
P-Casp-1-qRT	GAAATACTCCGAAGAAGTCCCAGA	GACCCCTTGCTTCTCACCAC
P-NLRP3-qRT	CCTCTTTGGCCTTGTAAACC	TGGCTGGGCTCAATCTGTAG
P-ASC-qRT	GCTGGCTAGCATGGGGTGCACGCGTGAC	GCCGCTCGAGTCAGCTCTGCTCCAGGTCG

P: porcine, h: human, m: mouse.

**Table 2:** PCR primers used to amplify the FMDV viral genes.

Genes	Sense Primers (5′-3′)	Anti-Sense Primers (5′-3′)
2B	ATGCCCTTCTTCTTCTCCGA	TCACTTGTCATCATCGTCC
VP3	ATGGGGATTTTCCCTGTGGCCTGTAG	TCACTTGTCATCATCGTCCTTATA
3A	ATGATCTCAATTCCTTCCCAA	TTGGGAAGGAATTGAGATCAT
VP2	ATGGATAAGAAAACCGAGGAG	CTCCTCGGTTTTCTTATCCAT
VP1	ATGACCACTTCGACGGGCGAG	CTCGCCCGTCGAAGTGGTCAT
3D	ATGGGGTTGATTGTGGACACC	GGTGTCCACAATCAACCCCAT
L	ATGAATACAACTGACTGTTTTATCGCT	TCATTTGAGCTTGCGTTGAACCTTGGC

## Data Availability

“MDPI Research Data Policies” at https://www.mdpi.com/ethics.
